# Supplementary material for: Electronic Janus lattice and kagome-like bands in coloring-triangular MoTe2 monolayers
Source: Nat Commun. 2023 Oct 9;14:6320. doi: 10.1038/s41467-023-42044-5 (PMC10562484; doi:10.1038/s41467-023-42044-5)
Supplement: Supplementary file 1 — Supplementary Information [file 41467_2023_42044_MOESM1_ESM.pdf]

Supplementary Materials for

Electronic Janus lattice and kagome-like bands in coloring-triangular $MoTe_2$ monolayers

Le Lei[1,2,+], Jiaqi Dai[1,2,+], Haoyu Dong [1,2,+], Yanyan Geng[1,2], Feiyue Cao[1,2], Cong Wang[1,2], Rui Xu[1,2], Fei Pang[1,2], Zheng-Xin Liu[1,2], Fangsen Li[3, 4], Zhihai Cheng[1,2, *], Guang Wang[5, 6, *] and Wei Ji[1,2, *]

[1]*Beijing Key Laboratory of Optoelectronic Functional Materials & Micro-nano Devices, Department of Physics, Renmin University of China, Beijing 100872, China*

[2]*Laboratory of Quantum State Construction and Manipulation (Ministry of Education), Renmin University of China, Beijing, 100872, China*

[3]*Vacuum Interconnected Nanotech Workstation, Suzhou Institute of Nano-Tech and Nano-Bionics, Chinese Academy of Sciences, Suzhou 215123, China*

[4]*School of Nano-Tech and Nano-Bionics, University of Science and Technology of China, Hefei 230026, China*

[5]*Department of Physics, College of Sciences, National University of Defense Technology, Changsha 410073, China*

[6]*State Key Laboratory of Low-Dimensional Quantum Physics, Department of Physics, Tsinghua University, Beijing 100084, China*

[+]These authors contributed equally: Le Lei, Jiaqi Dai, Haoyu Dong

*Email: zhihaicheng@ruc.edu.cn, wangguang@nudt.edu.cn, and wji@ruc.edu.cn

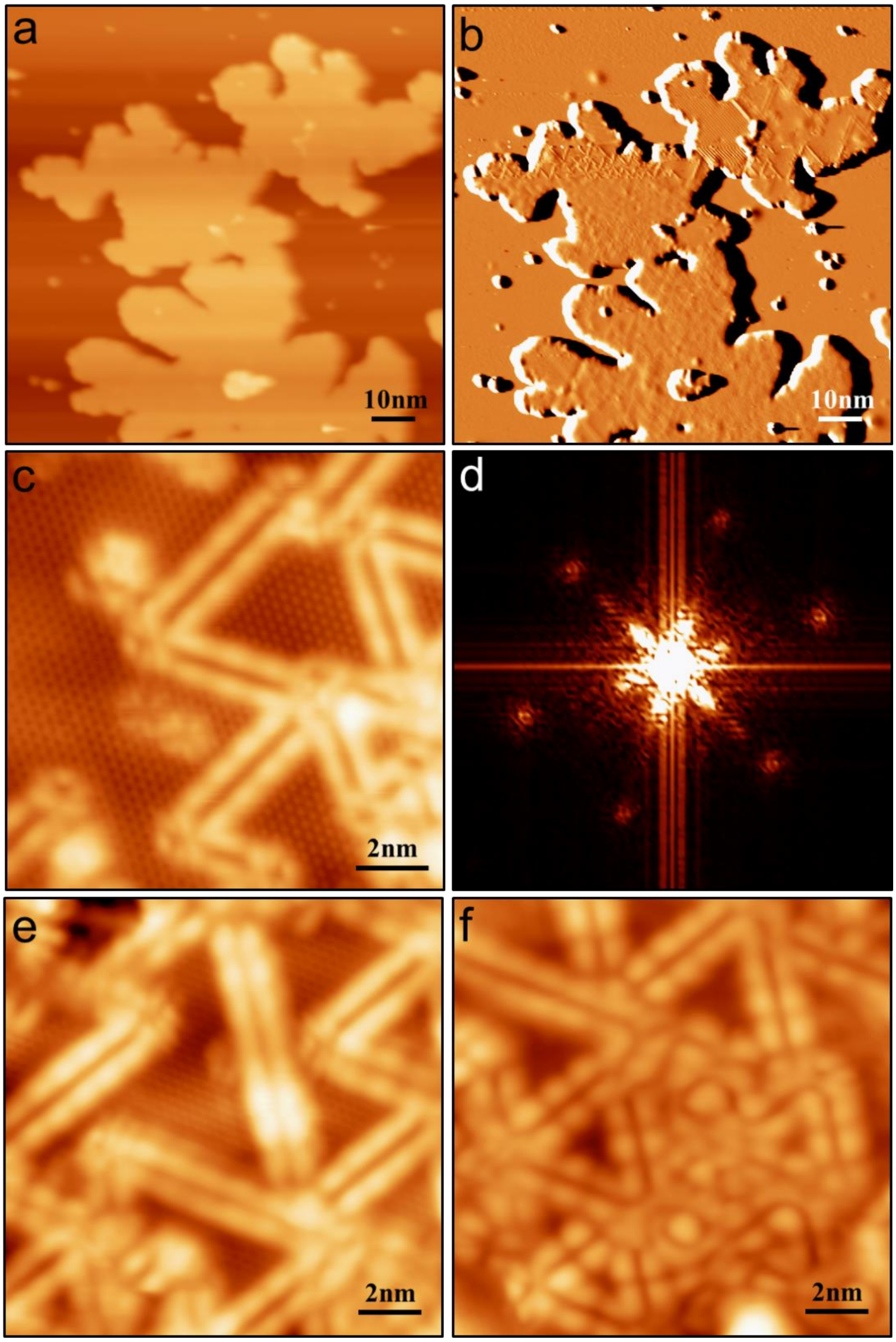

Supplementary Fig. 1. STM topographic images of the monolayer $MoTe_2$ after post-annealing at the growth temperature. (a) Large-scale STM topography image of monolayer 1H-$MoTe_2$ islands on HOPG substrate. (b) Corresponding STM current image of (a). (c) High-resolution STM topography image of monolayer 1H-$MoTe_2$, showing the 1×1 $MoTe_2$ domains and 1D MTBs. (d) Corresponding FFT image of (c). (e,f) High-resolution STM topography image of monolayer 1H-$MoTe_2$, showing triangle-shaped MTBs loops with different size. (a,b) V= 2.9V, I= 100pA; (c) V= -1.0V, I= -80pA; (e) V= -1.1V, I= -100pA; (f) V= -1.1V, I= -100pA.

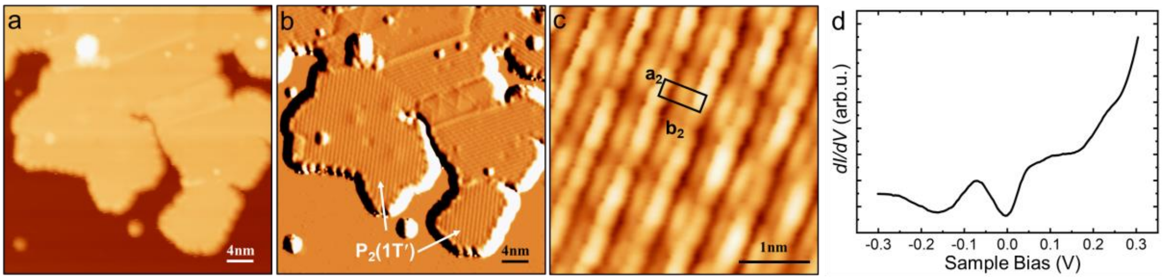

Supplementary Fig. 2. Large-scale STM topographic images of the monolayer $MoTe_2$ after post-annealing at the growth temperature. (a) Large-scale STM image of monolayer 1T'-$MoTe_2$ islands on HOPG substrate. (b) Corresponding STM current image of (a), showing clearly 1T' phase domains as marked by white arrows. (c) Atomic-resolution image of 1T'-$MoTe_2$. The unit cell is marked by rectangle. (d) Typical *dI/dV* spectra taken on the monolayer 1T'-$MoTe_2$. (a,b) V= 2.6V, I= 100pA; (c) V= -10mV, I= -1nA.

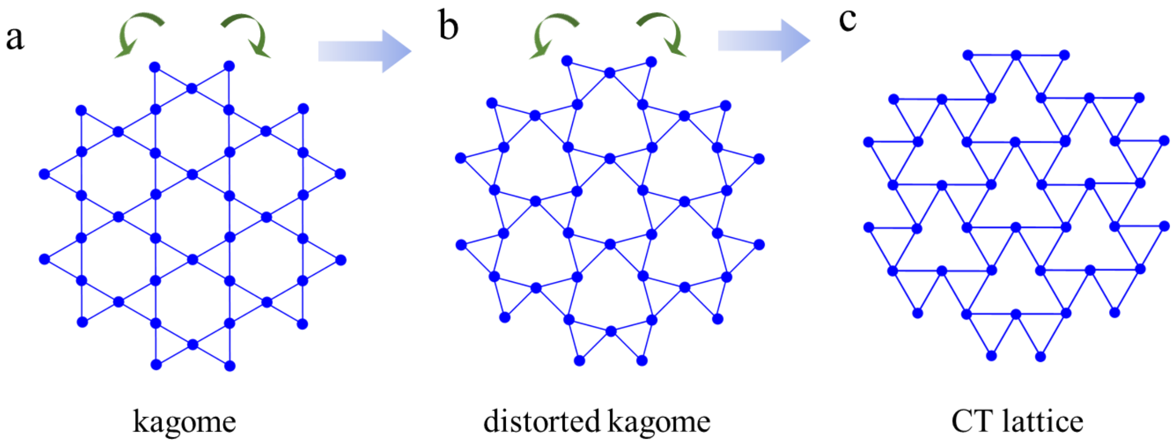

Supplementary Fig. 3. Line graph of kagome lattice and CT lattice. Line graph of a kagome lattice (a), distorted kagome lattice (b) and CT lattice (c). By rotating the triangles following the way illustrated in Supplementary Fig. 3a and b in the kagome lattice, one could obtain the CT lattice (Supplementary Fig. 3c). The CT-lattice, sharing with the kagome lattice, hosts a hexagonal lattice and a triangle on each of its nodes, which indicates the CT lattice is inherently equivalent to the kagome lattice. It has also been proven that the CT lattice is equivalent to the renowned kagome lattice mathematically by a unitary transformation [1]. The CT lattice Hamiltonian is shown to be connected to the Kagome lattice Hamiltonian through a unitary matrix of rotation operator, indicating that the two lattice Hamiltonians possess identical eigenvalues or band structures.

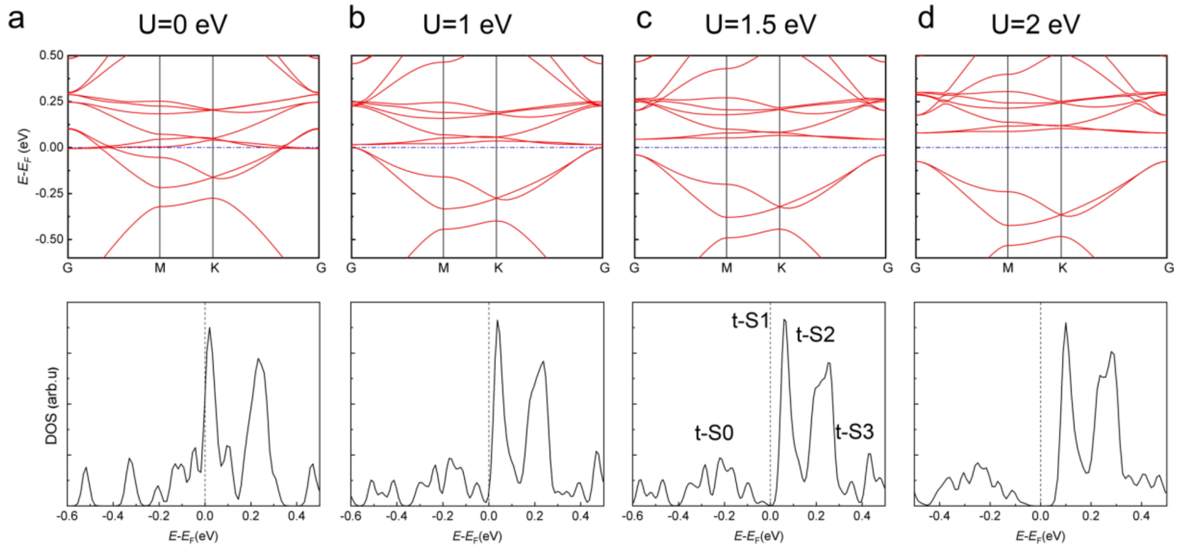

Supplementary Fig. 4. Electronic band-structure and DOSs with different Hubbard *U*. The *U* values are 0 eV(a), 1 eV (b), 1.5 eV (c) and 2 eV (d), respectively. In the upper panels, with the increase of *U*, CT1 bands move up while CT2 bands move down, bringing about a gradually increasing energy gap between t-S0 and t-S1 shown in the lower panels.

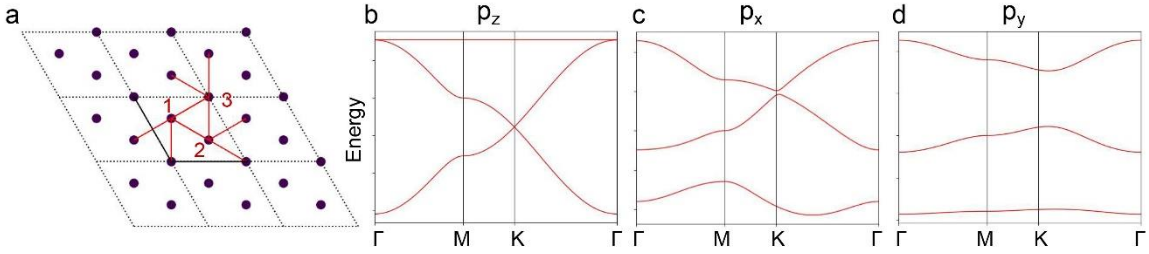

Supplementary Fig. 5. Single *p*-orbital in CT lattice. (a) Schematic plot of CT lattice [2] composed of Mo atoms. Orbitals are shown as filled circles and marked with red numbers, while hopping terms are shown as solid red lines. Thick black arrows denote the lattice vectors. (b-d) Band structure of CT lattice model, in which each atom carries one p_z (b), p_x (c), or p_y (d) orbital. Introduction of two- or even four-fold symmetric orbitals occupying CT-latticed sites may disturb the kagome band structures [3]. The p_z orbital case exhibits a perfect set of kagome band structures. However, the perfect kagome band structures degrade in the p_x or p_y case because the overlapping matrixes vary among different sites.

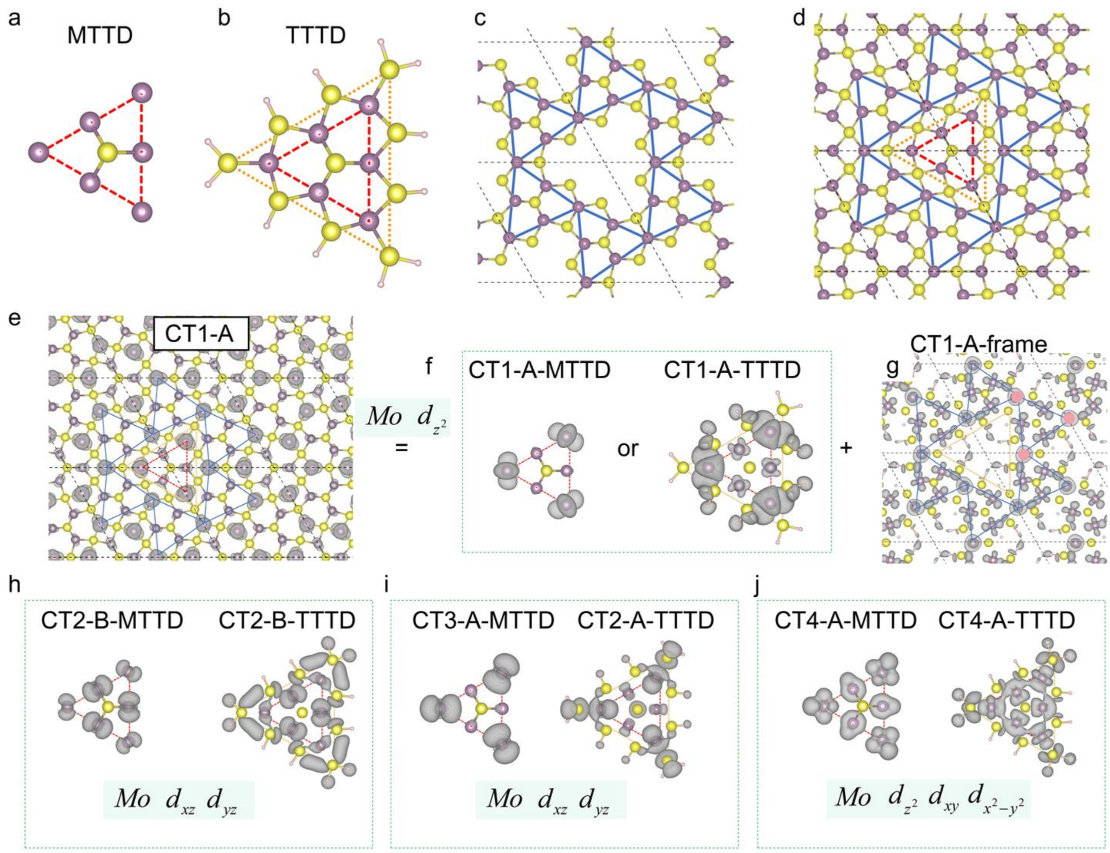

Supplementary Fig. 6. Geometric and electronic structures of CT lattice. (a-d) Atomic structures of (a) MTTD (highlighted by red dashed triangles), (b) TTTD (highlighted by orange dashed triangles). Their relations with that of the CT monolayer were illustrated in (c) MTB surrounding structures, referred to as frame and (d) CT-$MoTe_2$ (Mo_5Te_8) monolayer. (e-g) Isosurface contours of wavefunction norms for CT-$MoTe_2$ (Mo_5Te_8), TTTD and MTTD, and the frame. (e) CT1-A state of CT-$MoTe_2$ (Mo_5Te_8) at Γ point using an isosurface of 1×10^{-3} e $Bohr^{-3}$, mainly contributed by the d_{z^2} orbitals of Mo atoms on vertices of CT lattice and triangle domain. (f) show the calculated real-space distributions for CT1-A of TTTD and MTTD at Γ point. Isosurface is 5×10^{-3} e $Bohr^{-3}$. (g) show the distributions of MTB frame. Three CT sites in a primitive cell are shown as red filled circles. The d_{z^2} orbital dominates each CT sites for the MTB framework. (h)-(j) The same scheme of plots as that of panels (f) for CT2-B, CT3-A, and CT4-A, respectively, which depict their frontier orbitals. In light of both results, unlike those atomic p_x or p_y orbitals, introduction of these C_3 symmetrized orbitals for MTTD and/or TTTD does not change the original symmetry of the CT lattice, which leads to the kagome bands intrinsically originated from the symmetry of the CT lattice.

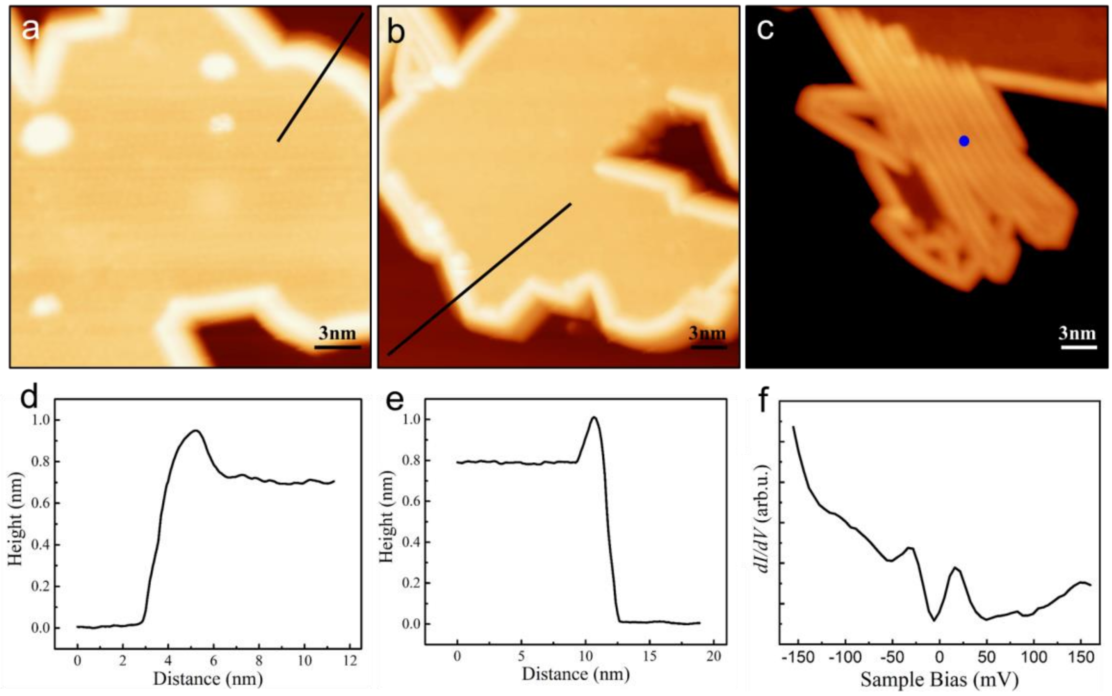

Supplementary Fig. 7. The edge-attached Mo_6Te_6 nanowires for the monolayer $MoTe_2$ islands on HOPG substrate after post-annealing at higher temperature. (a-c) STM topographic images of edge-attached Mo_6Te_6 nanowires. (d) The height profile along the black solid line in (a). (e) The height profile along the black solid line in (b). (f) Typical *dI/dV* spectra taken at the central locations of Mo_6Te_6 nanowire marked by blue dot in (c). (a-b) V= -2.0V, I= -100pA; (c) V=-1.5V, I= -100pA.

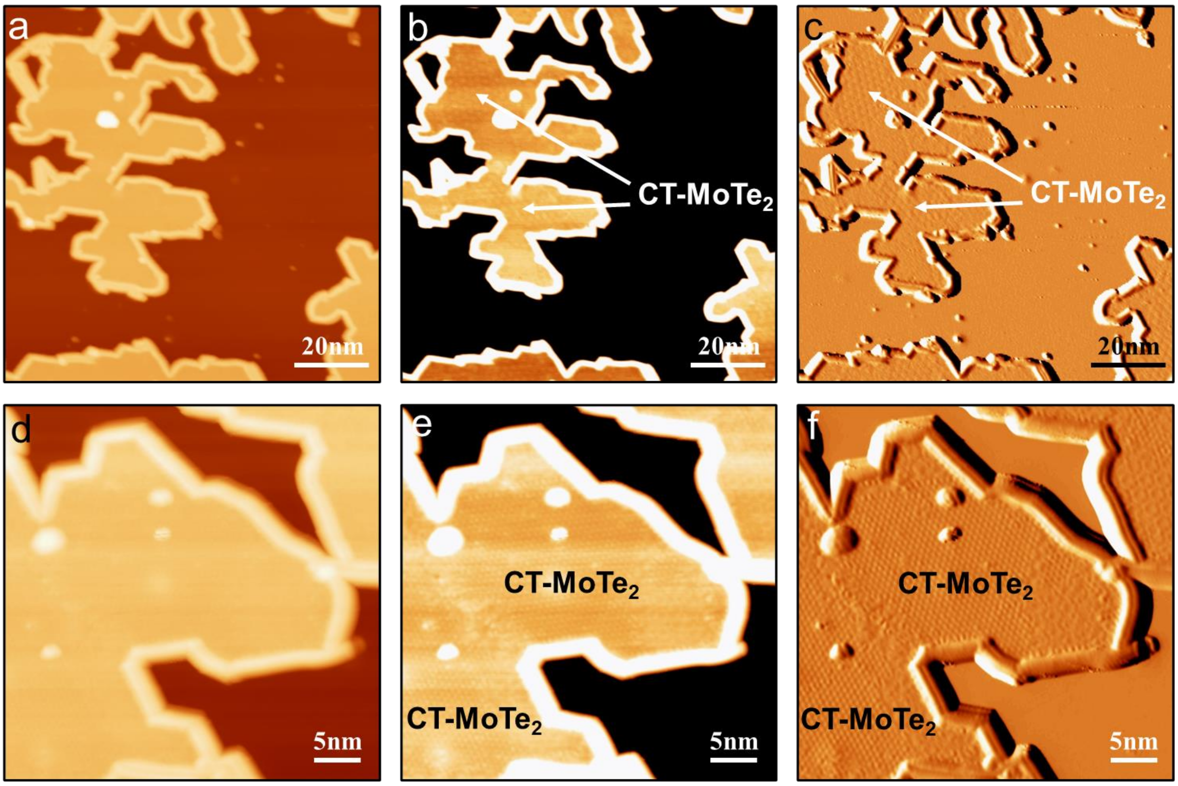

Supplementary Figure 8. Large-scale STM topography images of the CT-$MoTe_2$ monolayer after post-annealing at higher temperature. (a-b) Large-scale STM topography images of monolayer $MoTe_2$ islands on HOPG substrate with different contrast. (c) Corresponding STM current image of (a). (d-e) Large-scale STM topography images of monolayer CT-$MoTe_2$ islands on HOPG substrate with different contrast. (f) Corresponding STM current image of (d). (a-c) V= -2.2V, I= -100pA; (d-f) V= -2.0V, I= -100pA.

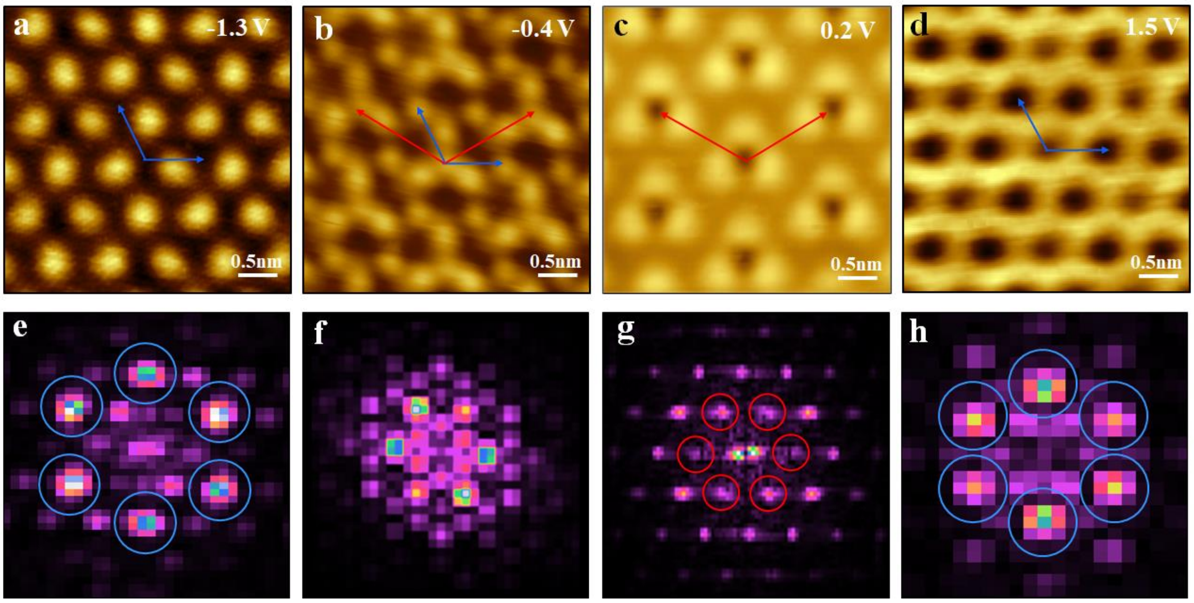

Supplementary Fig. 9. FFT of bias-dependent STM images of the CT-MoTe$_2$ phase. (a-d) Bias-dependent STM topography images of the CT-MoTe$_2$ phase, showing an apparent electronic Janus lattice. (e-h) The FFT images of corresponding STM image, which can show the atomic lattice and pseudo-sublattice, respectively. It is noted that the FFT points of the small pseudo-sublattice are more pronounced (highlighted by blue circles) in (e) and (h).

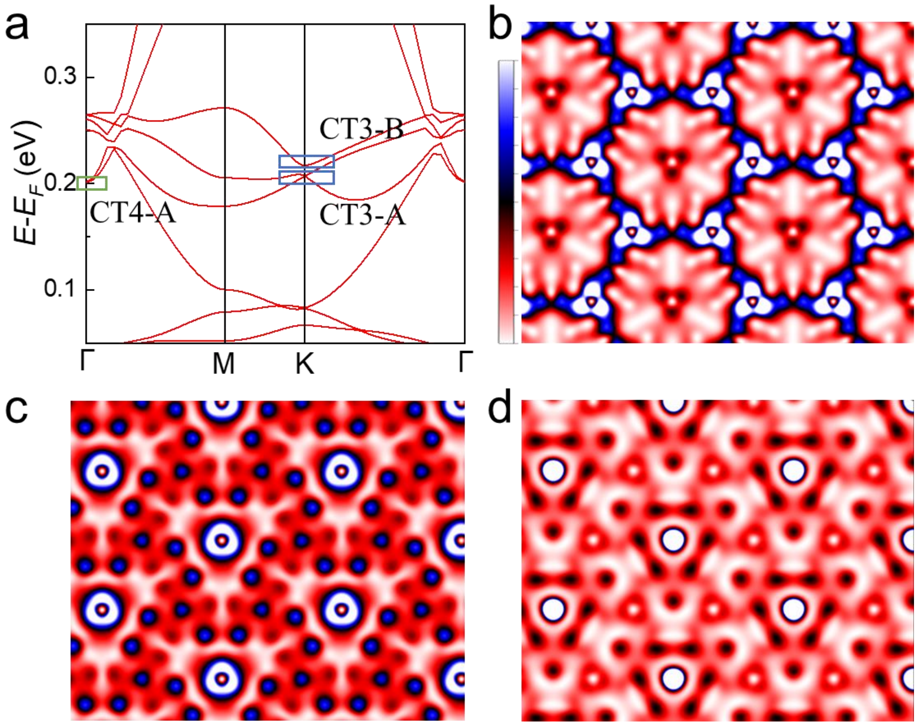

Supplementary Fig. 10. Electronic structures of CT3 and CT4 bands. (a) Calculated band structures. (b-d) Relative 2D contour of visualized wavefunction norms. Panel (b) plot CT4-A band at the Gamma point, marked by green rectangular box in (a). Panel (c) and (d) plot CT3-A and CT3-B bands at the K point, respectively, marked by blue rectangular boxes in (a).

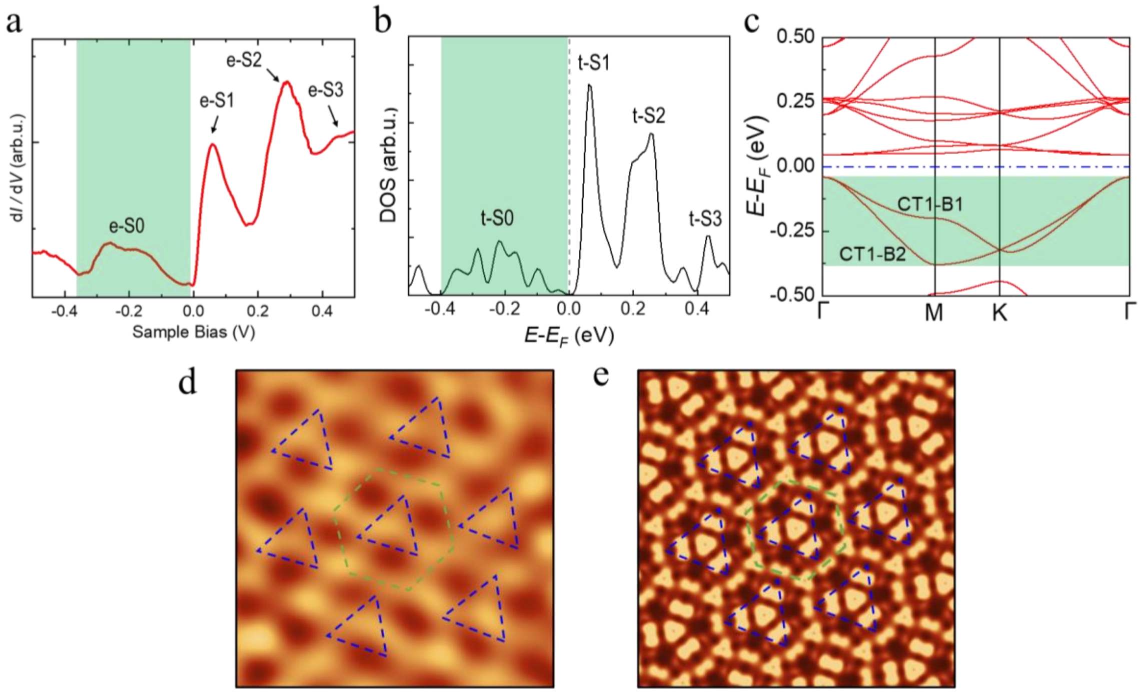

Supplementary Fig. 11. DFT-calculated LDOS and experimental *dI/dV* measurements of state S0 (-0.2V) in CT-$MoTe_2$ phase. (a,b) Magnified *dI/dV* spectrum of the in-gap states (a) and total DOS (b) of the CT-$MoTe_2$ phase (c) Theoretical band structures of the CT-$MoTe_2$ monolayer. The state S0 is highlighted by the green color in (a-c). (d) Constant-current *dI/dV* map at - 0.2 V. (e) Theoretically simulated maps derived from the wavefunction norms of the states sitting at -0.2eV.

State e-S0 corresponds to bands CT1-B1 and CT1-B2 in our DFT calculations. Bands CT1-B1 and CT1-B2 are ascribed to a Dirac band and a nominal “flat band”, respectively. Term “nominal” means that CT1-B2 is different from a usual flat band, which contacts CT1-B1 at the K point and is highly distorted and more dispersive. The *dI/dV* map exhibits two patterns, namely a triangular one (marked by blue triangles) being embedded in a distorted hexagonal one (marked by green hexagons), which are roughly consistent with the corresponding theoretical map. The hexagonal and embedding triangular patterns are also consistent with their origins from a Dirac state (CT1-B1) and a nominal “flat band” (CT1-B2). Because CT1-B2 is much more dispersive, the appearance of its associated DOS does not exhibit pronounced peak and the overall DOS appearance is dominated by the Dirac band (CT1-B1) showing a hexagonal pattern in the real-space.

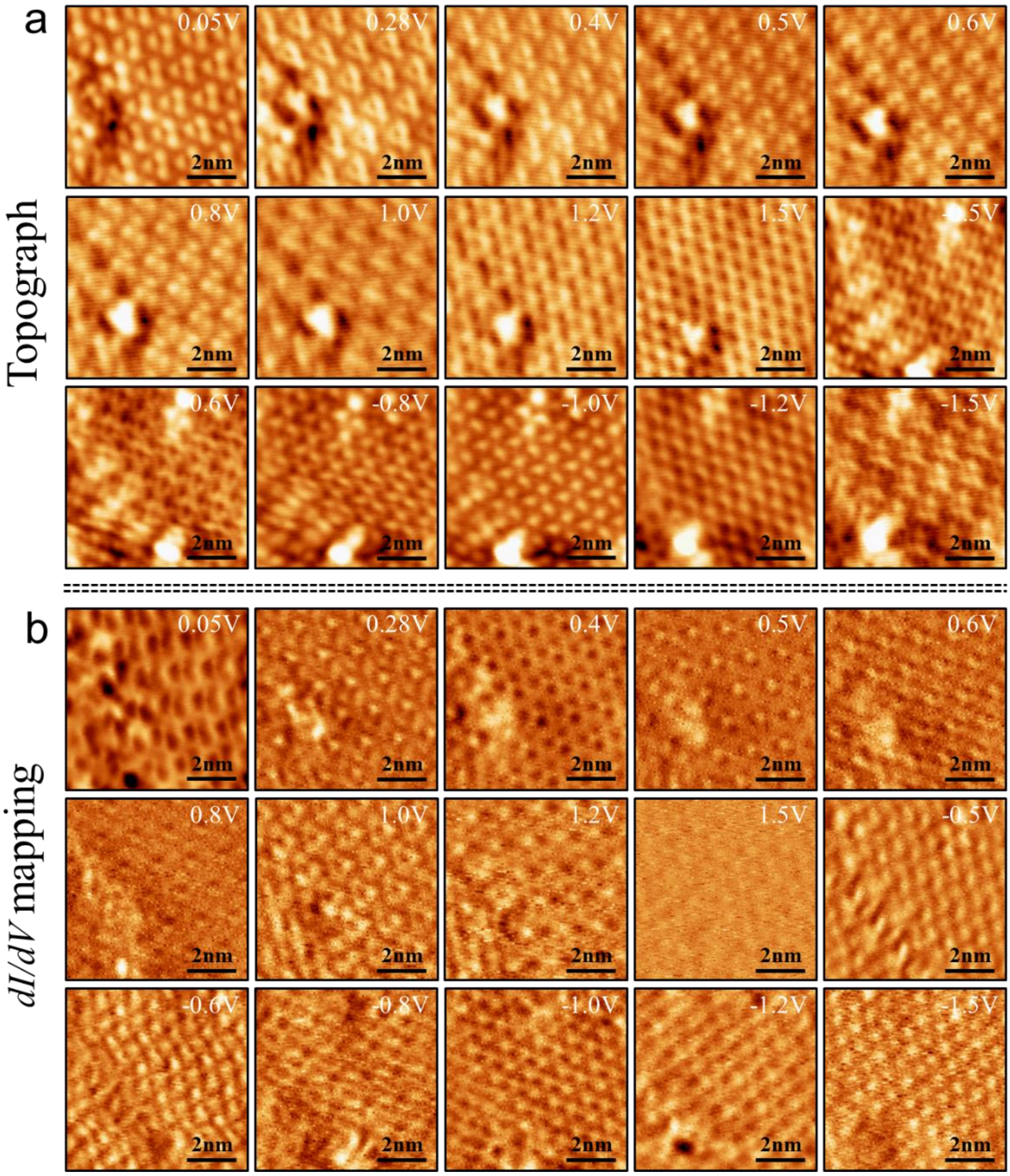

Supplementary Fig. 12. Scanning tunneling microscopy/spectroscopy measurement of the CT-$MoTe_2$. (a) Bias-dependent STM topographic images of CT-$MoTe_2$. (b) Corresponding *dI/dV* conductance maps of CT-$MoTe_2$.

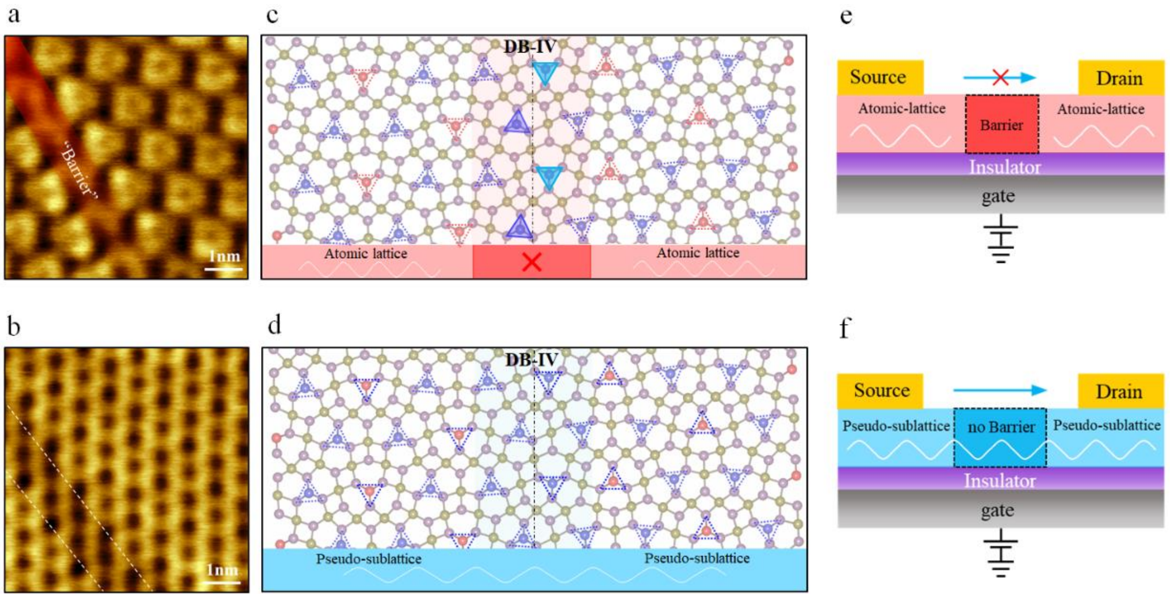

Supplementary Fig. 13. Schematic diagram of the domain boundary (DB-IV) assumed as the transport barrier or "transparent" for the charge carriers. (a,b) STM topography image of the translational symmetry broken in atomic-lattice (a) and the preserved translation symmetry in Te pseudo-sublattice (b). (c,d) Schematic atomic structure models of DB-IV in the atomic-lattice (c) and Te pseudo-sublattice (d). (e,f) Schematic of the DB-IV behave like a transport barrier (e, in the atomic-lattice) or "transparent" for the charge carriers (f, in the Te pseudo-sublattice).

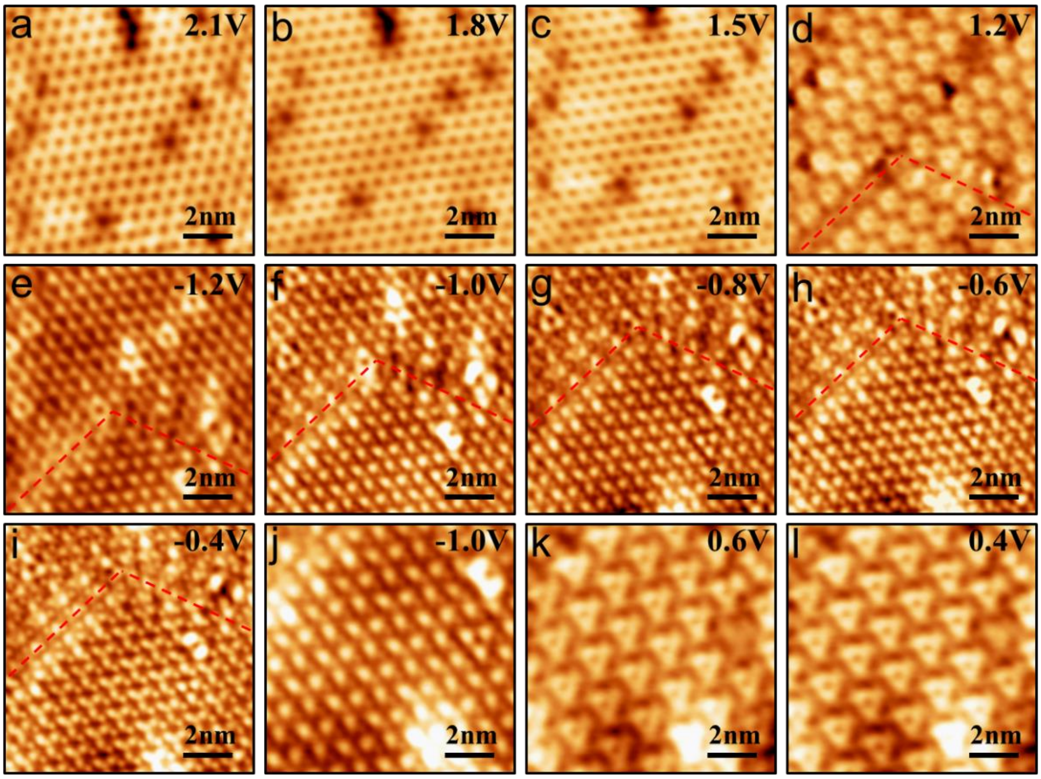

Supplementary Fig. 14. Bias-dependent STM topography images (a-l) of the DB-IV domain boundary in the CT-$MoTe_2$ monolayer. The domain boundary almost could not be resolved at (a-c) in the Te pseudo-sublattice of CT-$MoTe_2$. (a) V= 2.1V, I= 100pA; (b) 1.8V, I= 100pA; (c) V= 1.5V, I= 100pA; (d) V= 1.2V, I= 100pA; (e) V= -1.2V, I= -100pA; (f) V= -1.0V, I= -100pA; (g) V= -0.8V, I= -100pA; (h) V= -0.6V, I= -100pA; (i) V= -0.4V, I= -100pA; (j) V= -1.0V, I= -100pA; (k) V= 0.6V, I= 100pA; (l) V= 0.4V, I= 100pA. Note: The STM images are sequentially shifted due to the scanning drift. The domain boundary is highlighted by the red dashed lines.

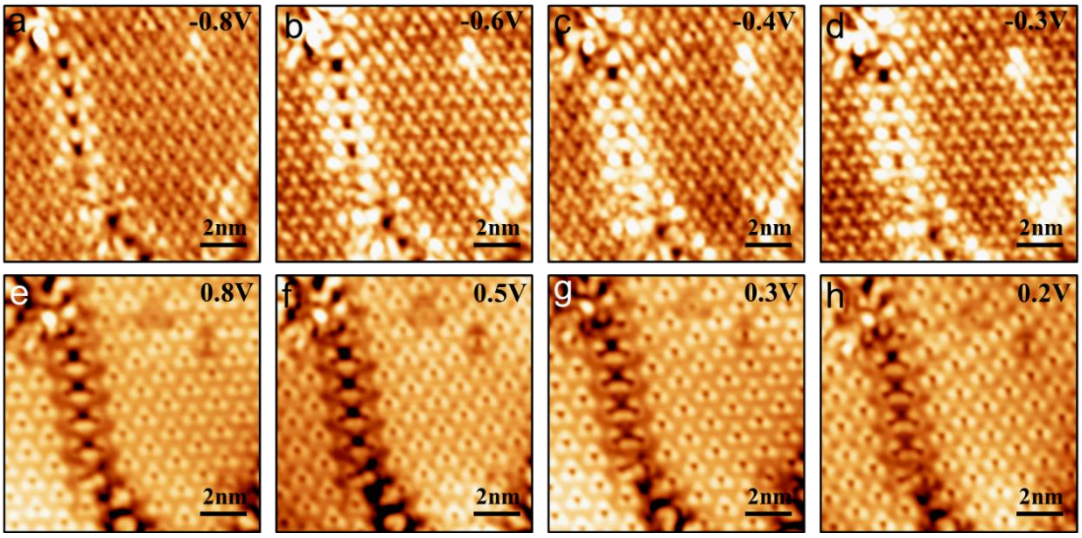

Supplementary Fig. 15. Bias-dependent STM topography images (a-h) of the DB-MT domain boundary within the CT-$MoTe_2$ monolayer. Domain boundary is clearly resolved within the intrinsic atomic-lattice. (a) V= -0.8V, I= -100pA; (b) -0.6V, I= -100pA; (c) V= -0.4V, I= -100pA; (d) V= -0.3V, I= -100pA; (e) V= 0.8V, I= 100pA; (f) V= 0.5V, I= 100pA; (g) V= 0.3V, I= 100pA; (h) V= 0.2V, I= 100pA.

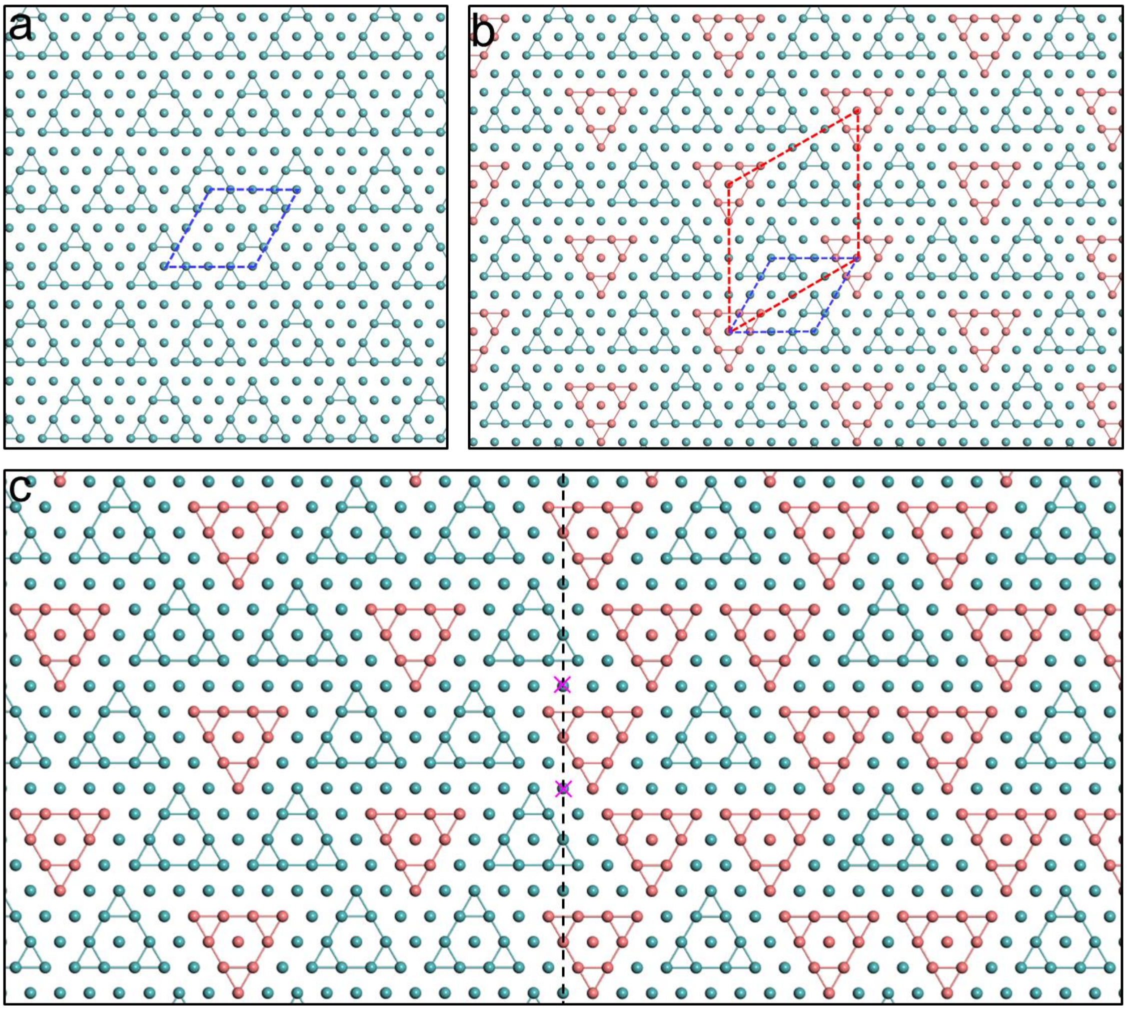

Supplementary Fig. 16. Schematic of the Possible polymorph and domain boundary in the 4×4 superstructure of TMDs. (a) The atomic structure of the 4×4 superstructure. (b) The atomic structure of polymorph in the 4×4 superstructure. (c) Atomic structural model of the designed DB-IV domain boundary. The inversion-symmetric centers at the domain boundary are marked by the crosses. All of the balls represent atoms of transition metal.

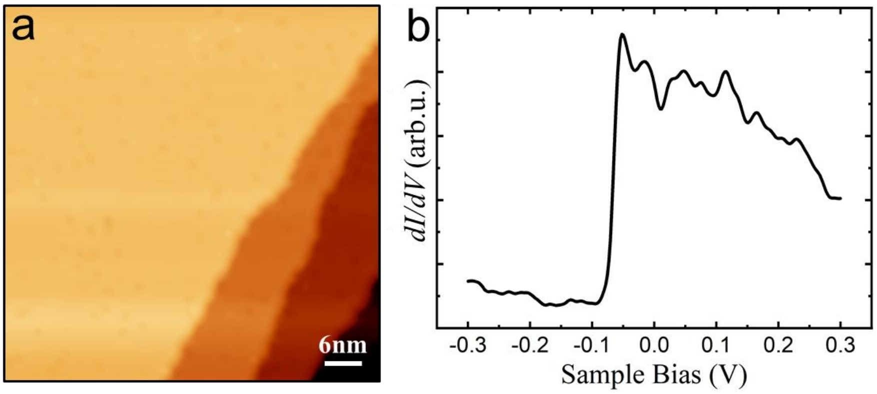

Supplementary Fig. 17. Spectral calibration of the STM tip on Ag(111) surface. (a) STM topographic image of Ag(111) surface. (b)Typical *dI/dV* spectrum measured on Ag(111) surface, which shows the Ag(111) surface state at V_s = **-** 60 mV. (a) V= 0.2V, I= 100pA.

Γ/K	1	2	3	4	5	6	7	8	9	10	11	12
Γ1/K1	1.00+0.00i	1.00+0.00i	1.00+0.00i	1.00+0.00i	1.00+0.00i	1.00+0.00i	1.00+0.00i	1.00+0.00i	1.00+0.00i	1.00+0.00i	1.00+0.00i	1.00+0.00i
Γ2/K2	1.00+0.00i	1.00+0.00i	1.00+0.00i	-1.00+0.00i	-1.00+0.00i	-1.00+0.00i	1.00+0.00i	1.00+0.00i	1.00+0.00i	-1.00+0.00i	-1.00+0.00i	-1.00+0.00i
Γ3/K3	1.00+0.00i	1.00+0.00i	1.00+0.00i	-1.00+0.00i	-1.00+0.00i	-1.00+0.00i	-1.00+0.00i	-1.00+0.00i	-1.00+0.00i	1.00+0.00i	1.00+0.00i	1.00+0.00i
Γ4/K4	1.00+0.00i	1.00+0.00i	1.00+0.00i	1.00+0.00i	1.00+0.00i	1.00+0.00i	-1.00+0.00i	-1.00+0.00i	-1.00+0.00i	-1.00+0.00i	-1.00+0.00i	-1.00+0.00i
Γ5/K5	2.00+0.00i	-1.00+0.00i	-1.00+0.00i	2.00+0.00i	-1.00+0.00i	-1.00+0.00i	0.00+0.00i	0.00+0.00i	0.00+0.00i	0.00+0.00i	0.00+0.00i	0.00+0.00i
Γ6/K6	2.00+0.00i	-1.00+0.00i	-1.00+0.00i	-2.00+0.00i	1.00+0.00i	1.00+0.00i	0.00+0.00i	0.00+0.00i	0.00+0.00i	0.00+0.00i	0.00+0.00i	0.00+0.00i
Γ7/K7	2.00+0.00i	-2.00+0.00i	-2.00+0.00i	0.00+0.00i	0.00+0.00i	0.00+0.00i	0.00+0.00i	0.00+0.00i	0.00+0.00i	0.00+0.00i	0.00+0.00i	0.00+0.00i
Γ8/K8	2.00+0.00i	1.00+0.00i	1.00+0.00i	0.00+0.00i	-1.73+0.00i	-1.73+0.00i	0.00+0.00i	0.00+0.00i	0.00+0.00i	0.00+0.00i	0.00+0.00i	0.00+0.00i
Γ9/K9	2.00+0.00i	1.00+0.00i	1.00+0.00i	0.00+0.00i	1.73+0.00i	1.73+0.00i	0.00+0.00i	0.00+0.00i	0.00+0.00i	0.00+0.00i	0.00+0.00i	0.00+0.00i

$D_{3h}(-6m2)$ Mult.	1	2	3	4	5	6	SYMMETRY OPERATIONS	1	2	3	4	5	6	7	8	9	10	11	12
	$A_1^{'}$	$A_1^{''}$	$A_2^{''}$	$A_2^{''}$	$E^{'}$	$E^{''}$		E	$2C_3$		σ_h	$2S_3$		$3C_2$			$3\sigma_v$		

Supplementary Table I. The character table of Γ/K -little group in space group 189. The atomic lattice of Mo_5Te_8 belongs to space group (SG) No. 189 and exhibits the D_{3h} point group which is the direct product of point group D_3 and a mirror symmetry operation σ_h. The six irreducible representations of D_{3h} and twelve symmetry operations are show in the bottom row of the table. The irreps calculations were performed using the IRVSP [4] program in conjunction with VASP. Γ1 to Γ6 represent six irreducible representations of D_{3h}: A_1'-E'', respectively; operations 1 to 12 represent twelve symmetry operations: E, $2C_3$, σ_h, $2S_3$, $3C_2$, and $3\sigma_v$.

Supplementary References

[1] Zhang, S. H. *et al*. Kagome bands disguised in a coloring-triangle lattice. *Phys. Rev. B* **99**, 100404 (2019).

[2] Li, Y. H. *et al*. TBPLaS: A tight-binding package for large-scale simulation. *Computer Physics Communications* **285,** 108632 (2023)

[3] Liu, H. *et al.* Orbital design of flat bands in non-line-graph lattices via line-graph wave functions. *Phys. Rev. B* **105**, 085128 (2022)

[4] Gao, J. C. *et al*. Irvsp: To obtain irreducible representations of electronic states in the VASP. *Comput. Phys. Commun.* **261**, 107760 (2021)
